# Social Class Inequalities in Self-rated Health and Their Gender and Age Group Differences in Japan

**DOI:** 10.2188/jea.16.223

**Published:** 2006-11-03

**Authors:** Kaori Honjo, Norito Kawakami, Tadashi Takeshima, Hisateru Tachimori, Yutaka Ono, Hidenori Uda, Yukihiro Hata, Yoshibumi Nakane, Hideyuki Nakane, Noboru Iwata, Toshiaki A. Furukawa, Makoto Watanabe, Yosikazu Nakamura, Takehiko Kikkawa

**Affiliations:** 1 Social and environmental medicine, Osaka University Graduate School of Medicine.; 2 Department of Mental Health, University of Tokyo Graduate School of Medicine.; 3 National Institute of Mental Health, National Center for Neurology and Psychiatry.; 4 Health Center, Keio University.; 5 Sensatsu Public Health Center.; 6 Oshima Hospital.; 7 Division of Human Sociology, Nagasaki International University Graduate School.; 8 Division of Neuropsychiatry, Department of Translational Medical Sciences, Nagasaki University Graduate School of Biomedical Sciences.; 9 Faculty of Human and Social Environment, Hiroshima International University.; 10 Department of Psychiatry and Cognitive-Behavioral Medicine, Nagoya City University Graduate School of Medical Sciences.; 11 Department of Public Health, Jichi Medical University.; 12 Chubu Gakuin University.

**Keywords:** Social Class, Health Status, Sex, Japan

## Abstract

**BACKGROUND:**

Few studies have examined social inequalities in self-rated health in Japan, and the issue of gender differences related to social inequalities in self-rated health remains inconclusive.

**METHODS:**

The data derived from interviews with 2987 randomly selected Japanese adults in four prefectures in Japan who completed the cross-national World Mental Health survey from 2002 through 2005. We calculated odds ratios (ORs) of having poor self-rated physical and mental health by two social class indicators independently with multivariate logistic regression models, adjusted for age, gender, marital status, and area. Stratified analyses by gender and age group were also conducted.

**RESULTS:**

The adjusted ORs of the lowest educational attainment category having poor self-rated physical and mental health were 1.42 (95% confidence interval [CI]: 1.15-1.76) and 1.37 (95% CI: 1.10-1.70), respectively. Among females, educational attainment had significant linear associations with self-rated physical and mental health. Adjusted household income was also significantly associated with self-rated physical health among female respondents. No associations were found among males. While educational attainment was associated with self-rated health among the young age group, adjusted household income was associated with self-rated physical health in the middle and old age group.

**CONCLUSION:**

These results indicated social inequalities in self-rated health and prominent social inequalities in self-rated health among females in Japan. Social inequalities in self-rated health seemed to exist across age groups. However, the mechanism of social inequalities in self-rated health could be different depending on the age group.

Japan demonstrates the longest life expectancy in the world: the life expectancies at birth in Japan were estimated to be 78.4 years for males and 85.3 years for females in 2003.^1^ In addition to the improvement of living conditions due to economic growth since the early 1960s, several researchers have noted that, compared to other countries, the relatively smaller social disparities in Japanese society may be a reason for this longevity.^2^^,^^3^ However, the relatively egalitarian Japanese society has been changing; the gap between social classes has been increasing since the late 1980s,^4^ and social inequalities with respect to the health of Japanese society are now becoming a concern.

The relationship between social class and health status is a well-established finding in epidemiologic research,^5^^-^^12^ which consistently shows that people from higher social classes have lower morbidity and mortality from various diseases, illnesses, and health problems, compared to those from lower social classes.^13^^-^^17^

Social inequalities in self-rated health are also well documented; people from lower social classes rate their health poorer compared to those from higher social classes.^18^^-^^21^ In terms of gender differences in social inequalities of self-rated health, several previously conducted studies have shown that the magnitudes of social class inequalities in self-rated health differ between males and females, with shallower or more inconsistent gradients found among females than males.^20^^-^^22^ However, some studies show that males and females have a similar pattern of social inequality in self-rated health. Marmot et al^23^ demonstrated a similar social gradient in self-rated health and psychological wellbeing between genders. Moreover, Matthews et al^24^ indicated that there was no consistent evidence for greater gradients in self-rated health for men; rather, they suggested that the magnitude of social inequalities was greater for women with poor self-rated health at age 23 years and psychological distress at age 33. Clearly, the issue of gender differences with respect to the social inequalities of self-rated health is inconclusive and requires further research.

Social inequalities in self-rated health are well documented in Europe and the United States.^18^^,^^25^ However, few studies have been conducted to examine them in Japan. Shibuya et al^26^ showed that household income adjusted by the number of family members had an independent association with self-rated health, adjusting for income distribution at the prefecture level, and ecological and individual level covariates. They did not, however, examine gender differences in social inequalities in self-rated health. A study conducted by Martikainen et al^20^ indicated socioeconomic differences in self-rated health among Japanese employees. They found an inverse association between self-rated health and employment grade among Japanese male employees, while among Japanese female employees they concluded that there were small and inconsistent differences in self-rated health by employment grade. Nishi et al^21^ demonstrated an inverse association of self-rated health by employment grade in both male and female Japanese civil servants; however, educational attainment level was a significant predictor for self-rated health only in male employees. Although still limited, the above evidence suggests that social class gradients in self-rated health exist in Japan and that social inequalities in self-rated health seem to be more meaningful among males than females.

According to the national data,^27^ it is clear that Japan has been drastically moving toward the popularization of higher education over the last few decades. As people became eager to attain higher education, the range of educational attainment levels has become wide and social inequalities have deepened in Japan.^4^ Considering such drastic social changes over the last few decades, social inequalities in self-rated health in Japan should be greater in the younger generation than in older generations as social inequality becomes more manifest.

In this study, we examined the association between self-rated health and social class using educational attainment and household income adjusted for household size independently in random samples from four selected prefectures in Japan. Our aims were: (1) to test the association between relevant social class indicators (educational attainment and adjusted household income) and self-rated physical and mental health; (2) to examine gender differences with respect to the social inequalities of self-rated health; and (3) to examine the age group influence on social inequalities in self-rated health. We hypothesized that people in lower social classes were more likely to report poor self-rated physical and mental health than those in higher social classes, that social inequalities in self-rated health were greater among males than females, and that social inequalities in self-rated health were greater in the younger generation compared to older generations.

## METHODS

### Survey Populations and Study Sample

The data derive from face-to-face interviews of Japanese adults in Japan collected as part of the cross-national World Mental Health (WMH) survey conducted in 28 countries around the world. In Japan, based on the availability of site investigators and the cooperation of local governments, eight sites in four prefectures were selected as study sites between 2002 and 2005: Okayama Prefecture (the cities of Okayama and Tamano), Nagasaki Prefecture (the city of Nagasaki), Kagoshima Prefecture (the city of Kushikino, and the towns of Fukiage, Ichiki, and Higashiichiki), and Tochigi Prefecture (the city of Sano). The WMH Japan surveys were conducted with a probability sample of adult residents 20 years of age and older at each survey site, based on voter registration lists or resident registries.

Trained interviewers initially contacted 5622 subjects and then interviewed 3517 subjects who agreed to participate in the survey. We excluded 519 subjects who did not meet eligibility criteria: those who had died, moved, or were institutionalized. Eleven subjects did not complete the interview. The sample for the present analysis was drawn from the 2987 respondents who completed the survey from November 2002 through March 2005. The overall response rate (the number of completed interviews divided by the number of eligible subjects) was 58.5 %. In addition, the subjects who did not complete the interview were more likely to be younger than our study subjects, and males were less likely to complete the interviews than females.

The procedures were fully explained to respondents, and written consent was obtained from each respondent at each site. The study was approved by the Committees of Ethics in Research of Human Subjects at Okayama University (for the Okayama site), at Japan National Institute of Mental Health (for the Kagoshima site), at Nagasaki University (for the Nagasaki site), and at Jichi Medical School (for the Tochigi site). The sampling design and procedures have been described in further detail elsewhere.^28^

### Measurements

The primary independent variable in this study was social class. We independently used educational attainment and household income adjusted by the number of family members as indicators of social class.

The respondents were categorized into three groups according to the duration of education: 13 years or longer (0), 12 years (1), and 11 years or shorter (2). Household income was estimated by the total of before-tax personal income, the partner’s income, other family members’ incomes, public pensions, government assistance, and income from other sources in the past year. As all questions on income were asked using categories, we assigned income values based on the mid-point of each category. We adjusted household income for household size with an equivalence elasticity of 0.5, as used in previous studies.^26^^,^^29^^,^^30^ All respondents were categorized into four groups by adjusted household income: (0) highest group, (1) second highest group, (2) second lowest group, and (3) lowest group.

Self-rated health is widely used and there is extensive evidence that it predicts mortality and morbidity. It has been shown to be not only strongly associated with a variety of indicators of well-being^31^ but also a strong predictor of mortality in longitudinal studies.^32^ Respondents were asked to rate their general physical health on a five-point scale ranging from excellent to poor. We grouped this rating into a binary variable of poor physical health (“poor” and “fair”) and good physical health (“good,” “very good,” and “excellent”). Good physical health was the reference group. Similarly, self-rated mental health was grouped into a binary variable of poor mental health (“poor” and “fair”) and good mental health (“good,” “very good,” and “excellent”). Good mental health was the reference group.

Age was measured in years and categorized into three groups: 20-40, 41-60, and 61 and older. Gender was treated as a bivariate variable; the reference group was male. Marital status was categorized into three groups: married, separated/widowed/divorced, and never married.

### Data Analysis

We examined the social inequalities in self-rated physical health and self-rated mental health, hypothesizing that people in lower social classes would have higher odds of having poor self-rated physical and mental health compared to those in higher social classes. We used chi-square tests to estimate bivariate associations between social class (educational attainment and adjusted household income) and other covariates with health outcomes.

Logistic regression analyses were used to estimate odds ratios (ORs) of having poor self-rated physical and mental health by two social class indicators (educational attainment and adjusted household income). We entered social class indicators as categorical variables first and then added the covariates to the models, regardless of their statistical significance. The covariates based on theory are age in years, gender, marital status, and area. We calculated the adjusted OR and 95% confidence interval (CI) of having poor self-rated physical and mental health by the two social class indicators (educational attainment and adjusted household income) independently. A test for linear trend was performed for each model. We also conducted a stratified analysis by gender for both outcomes in order to accomplish our second aim as well as a stratified analysis by age group in order to achieve our third aim. The standard errors for the ORs were calculated using the Wald test.^33^ All analyses were conducted with the SAS^®^ statistical package.^34^

## RESULTS

Table 1 represents the selected characteristics of the study population, which consisted of Japanese adults in Japan 20 years of age and older who completed the survey (n=2987). Forty-eight percent (n=1443) of the respondents reported poor physical health, while 41% (n= 1234) reported poor mental health. Both the mean and median ages were 54 years. Fifty-six percent of the respondents were female. Approximately 72% of the respondents were married, while 13% had never married. Both the mean and median years of educational attainment were 12 years. Thirty percent of the respondents had received education for equal to or shorter than 11 years, while 34% had received education for 13 years or longer. Forty-three percent of the respondents were from the Okayama site, while 7% were from the Nagasaki site. The proportion of respondents who had poor self-rated physical health varied significantly by age group, marital status, educational attainment, adjusted household income, and area. There was no statistically significant association between self-rated physical health and gender. Similarly, the proportion of respondents who reported poor self-rated mental health varied significantly by age group, gender, marital status, adjusted household income and area, while there was no statistically significant association between self-rated mental health and educational attainment.

**Table 1.** Characteristics of survey sample in the WHO World Mental Health Japan Survey 2002-2003 (n=2987).

	n(%)	Poor physical health		Poor mental health	
		1443(48.3%)	p-value	1234(41.3%)	p-value
Age (mean/median: 54 years)					
20-40 years	739 (24.8)	254 (34.4)	<0.0001	280 (37.9)	0.03
41-60	1166 (39.0)	571 (49.0)		511 (43.9)	
61+	1082 (36.2)	618 (57.1)		443 (40.9)	

Gender					
Male	1314 (44.0)	656 (49.9)	n.s.	519 (39.5)	0.07
Female	1673 (56.0)	787 (47.1)		715 (42.8)	

Marital status					
Married	2142 (71.7)	1033 (48.2)	<0.0001	860 (40.2)	0.01
Divorced/Separated/Widowed	456 (15.3)	254 (55.7)		217 (47.6)	
Never married	388 (13.0)	155 (40.0)		156 (40.2)	

Education (years of education)	(mean/median: 12)				
13 years or longer	945 (34.0)	387 (41.0)	<0.0001	357 (37.8)	n.s.
12 years	976 (35.2)	446 (45.7)		402 (41.2)	
11 years or shorter	854 (30.8)	486 (56.9)		357 (41.8)	

Adjusted household income					
Highest group	550 (24.5)	248 (45.1)	0.006	217 (39.5)	0.04
2nd highest	574 (25.6)	249 (43.4)		216 (37.6)	
2nd lowest	560 (25.0)	281 (50.2)		233 (41.6)	
Lowest group	557 (24.9)	293 (52.6)		255 (45.8)	

Area					
Okayama	1274 (42.7)	583 (45.8)	0.025	505 (39.6)	0.007
Kagoshima	955 (32.0)	472 (49.5)		382 (40.0)	
Nagasaki	208 (7.0)	117 (56.3)		106 (51.2)	
Tochigi	548 (18.4)	270 (49.3)		241 (44.0)	

Table 2 shows the adjusted ORs of having poor self-rated health by educational attainment and adjusted household income along with the *p* values for the linear trend test for each model. Controlling for age, gender, marital status, and area, the ORs of the lowest educational attainment category having poor self-rated physical and mental health were 1.42 (95% CI: 1.15-1.76) and 1.37 (95% CI: 1.10-1.70), respectively. The associations between two social class indicators (educational attainment and adjusted household income) and self-rated physical health were significantly linear (p=0.04 and p=0.01, respectively).

**Table 2.** Adjusted* odds ratios (ORs) and their 95% confidence intervals (CIs) for poor self-rated physical and mental health according to social class indicators (educational attainment and adjusted household income) and p values for trend test stratified by gender.

	All (n=2986)			Male (n=1314)			Female (n=1673)		
		
	n (%)	OR (95% CI)	p^†^	n (%)	OR (95% CI)	p^†^	n (%)	OR (95% CI)	p^†^
Self-rated physical health									
Educational attainment model									
13 years or longer	945 (34.0)	1.00	0.04	454 (37.2)	1.00	n.s.	491 (31.6)	1.00	0.02
12 years	976 (35.2)	1.10 (0.91-1.33)		417 (37.2)	1.07 (0.82-1.41)		559 (36.0)	1.15 (0.89-1.49)	
11 years or shorter	854 (30.8)	1.42 (1.15-1.76)		350 (26.7)	1.21 (0.89-1.64)		504 (32.4)	1.63 (1.21-2.21)	

Adjusted household income model									
Highest	550 (24.5)	1.00	0.01	294 (29.3)	1.00	n.s.	256 (20.7)	1.00	<0.001
2nd highest	574 (25.6)	0.95 (0.75-1.21)		293 (29.3)	0.90 (0.65-1.25)		281 (22.7)	1.02 (0.71-1.44)	
2nd lowest	560 (25.0)	1.21 (0.95-1.54)		237 (23.7)	1.04 (0.78-1.57)		323 (26.1)	1.38 (0.98-1.95)	
Lowest	557 (24.9)	1.23 (0.98-1.55)		177 (17.7)	0.92 (0.66-1.28)		380 (30.7)	1.57 (1.14-2.17)	

Self-rated mental health									
Educational attainment model									
13 years or longer	945 (34.1)	1.00	0.22	454 (37.2)	1.00	n.s.	491 (31.7)	1.00	0.04
12 years	975 (35.2)	1.17 (0.97-1.41)		417 (34.1)	1.11 (0.84-1.48)		558 (35.9)	1.22 (0.95-1.58)	
11 years or shorter	854 (30.8)	1.37 (1.10-1.70)		350 (28.7)	1.29 (0.95-1.76)		504 (32.5)	1.46 (1.08-1.97)	

Adjusted household income model									
Highest	549 (24.5)	1.00	0.12	294 (29.4)	1.00	n.s.	255 (20.6)	1.00	0.004
2nd highest	574 (25.6)	0.92 (0.73-1.18)		293 (29.3)	0.86 (0.61-1.21)		281 (22.7)	0.98 (0.69-1.39)	
2nd lowest	560 (25.0)	1.09 (0.85-1.39)		237 (23.7)	1.07 (0.75-1.53)		323 (26.1)	1.11 (0.80-1.56)	
Lowest	557 (24.9)	1.13 (0.90-1.42)		177 (17.7)	1.11 (0.80-1.55)		380 (30.7)	1.14 (0.83-1.56)	

* : all ORs were adjusted by age, marital status, and area.

† : p value for trend test

We found no significant associations between social class indicators and self-rated physical and mental health among male respondents, while we identified significant social inequalities in self-rated physical and mental health among female respondents (Table 2). The ORs of the lowest educational attainment category having poor self-rated physical and mental health among female respondents were 1.63 (95% CI: 1.21-2.21) and 1.46 (95% CI: 1.08-1.97), respectively. The ORs of the second lowest and the lowest adjusted household income categories having poor self-rated physical health among female respondents were 1.38 (95% CI: 0.98-1.95) and 1.57 (95% CI: 1.14-2.17), respectively.

In order to explore the mechanisms of gender differences with respect to social inequalities in self-rated health, we conducted a further stratified analysis by employment situation in addition to gender (Table 3). The ORs of the lowest educational category having poor self-rated physical and mental health among female workers were 1.72 (95% CI: 1.19-2.50) and 1.43 (95% CI: 1.00-2.07), respectively. The ORs of the lowest and second lowest adjusted household income groups having poor self-rated physical health were 1.55 (95% CI: 1.05-2.30) and 1.59 (95% CI: 1.03-2.46), respectively. The associations between educational attainment level and self-rated physical and mental health among housewives were similar to those among employed females, although they were not significant. However, adjusted household income seemed to be associated with only self-rated physical health and not with mental health among housewives. We identified no significant association between social class and self-rated health among male respondents stratified by employment situation (Not shown in the table).

**Table 3.** Adjusted* odds ratios (ORs) and their 95% confidence intervals (CIs) for poor self-rated physical and mental health according to social class indicators (educational attainment and adjusted household income) and p values for trend test among females stratified by employment situation.

	Employment situation								
	Housewife (n=601)			Worker (n=1010)			Retired (n=62)		
		
	n (%)	OR (95% CI)	p^†^	n (%)	OR (95% CI)	p^†^	n (%)	OR (95% CI)	p^†^
Self-rated physical health									
Educational attainment model									
13 years or longer	115 (19.6)	1.00	0.22	364 (40.2)	1.00	0.14	12 (20.0)	1.00	n.s.
12 years	189 (32.1)	1.30 (0.79-2.13)		351 (38.7)	1.19 (0.77-1.46)		19 (31.7)	1.16 (0.22-6.01)	
11 years or shorter	284 (48.3)	1.54 (0.87-2.75)		191 (21.1)	1.72 (1.19-2.50)		29 (48.3)	1.00 (0.14-7.02)	

Adjusted household income model									
Highest	64 (13.7)	1.00	0.13	191 (26.2)	1.00	0.03	1 (2.5)	not applicable	
2nd highest	82 (17.6)	1.26 (0.64-2.47)		191 (26.2)	0.91 (0.59-1.38)		8 (20.0)		
2nd lowest	142 (30.5)	1.10 (0.59-2.03)		168 (22.9)	1.59 (1.03-2.46)		13 (32.5)		
Lowest	178 (38.2)	1.58 (0.87-2.85)		184 (25.7)	1.55 (1.05-2.30)		18 (45.0)		

Self-rated mental health									
Educational attainment model									
13 years or longer	115 (19.6)	1.00	0.20	364 (40.2)	1.00	0.55	12 (20.0)	1.00	n.s.
12 years	189 (32.1)	1.28 (0.77-2.13)		350 (38.7)	1.21 (0.90-1.65)		19 (31.7)	1.13 (0.21-6.308)	
11 years or shorter	284 (48.3)	1.42 (0.78-2.58)		191 (21.1)	1.43 (1.00-2.07)		29 (48.3)	1.13 (0.21-9.98)	

Adjusted household income model									
Highest	64 (13.7)	1.00	n.s.	190 (25.9)	1.00	0.04	1 (2.5)	not applicable	
2nd highest	82 (17.6)	0.84 (0.43-1.66)		191 (26.6)	1.00 (0.66-1.52)		8 (20.0)		
2nd lowest	142 (30.5)	0.81 (0.44-1.50)		168 (22.9)	1.39 (0.90-2.13)		13 (32.5)		
Lowest	178 (38.2)	0.82 (0.45-1.49)		184 (25.1)	1.39 (0.94-2.05)		18 (45.0)		

* : all ORs were adjusted by age, marital status, and area.

† : p value for trend test

Age group influence on the association between social class and self-rated health was identified by stratified analysis by age group (Table 4). Among respondents who were between 20 and 40 years old, the OR of the lowest educational attainment category having poor self-rated physical health was 1.96 (95% CI: 1.18-3.24), and the ORs of the lowest and the second lowest educational attainment categories having poor self-rated mental health were 1.85 (95% CI: 1.17-3.05) and 1.46 (95% CI: 1.04-2.05), respectively. Among respondents who were between 41 and 60 years of age, the ORs of the lowest and the second lowest adjusted household categories having poor self-rated physical health were 1.26 (95% CI: 0.91-1.75) and 1.61 (95% CI: 1.11-2.34), respectively. Associations between educational attainment level and self-rated health were also identified but were not significant. Among those 61 years old and older, the OR of the lowest adjusted household income group with poor self-rated physical health was 1.57 (95% CI: 1.01-2.45). The association between both social class indicators (educational attainment and household income) and self-rated physical health was significantly linear (p=0.04, p=0.003, respectively).

**Table 4.** Adjusted* odds ratios (ORs) and their 95% confidence intervals (CIs) for poor self-rated physical and mental health according to social class indicators (educational attainment and adjusted household income) and p values for trend test for all respondents and stratified analysis by age groups.

	Age (year)								
	20-40 (n=739)			41-60 (n=1165)			61 and older (n=1082)		
		
	n (%)	OR (95% CI)	p^†^	n (%)	OR (95% CI)	p^†^	n (%)	OR (95% CI)	p^†^
Self-rated physical health									
Educational attainment model									
13 years or longer	409 (60.2)	1.00	n.s.	405 (36.9)	1.00	0.13	131 (13.2)	1.00	0.04
12 years	244 (35.9)	0.88 (0.62-1.26)		485 (44.1)	1.28 (0.97-1.67)		247 (24.8)	0.76 (0.49-1.17)	
11 years or shorter	27 (4.0)	1.96 (1.18-3.24)		209 (19.2)	1.28 (0.91-1.79)		618 (62.1)	1.32 (0.88-1.98)	

Adjusted household income model									
Highest	83 (16.2)	1.00	n.s.	350 (35.6)	1.00	0.07	117 (15.7)	1.00	0.003
2nd highest	145 (28.3)	0.64 (0.36-1.14)		177 (27.4)	1.16 (0.84-1.60)		160 (21.5)	0.94 (0.58-1.53)	
2nd lowest	149 (29.1)	1.04 (0.59-1.84)		187 (18.0)	1.61 (1.11-2.34)		234 (31.4)	1.14 (0.72-1.81)	
Lowest	135 (26.4)	0.93 (0.53-1.62)		187(19.0)	1.26 (0.91-1.75)		235 (31.5)	1.57 (1.01-2.45)	

Self-rated mental health									
Educational attainment model									
13 years or longer	409 (60.2)	1.00	0.17	405 (36.9)	1.00	0.56	131 (13.2)	1.00	0.25
12 years	244 (35.9)	1.46 (1.04-2.05)		484 (44.1)	1.13 (0.86-1.48)		247 (24.8)	0.76 (0.49-1.18)	
11 years or shorter	27 (4.0)	1.85 (1.17-3.05)		209 (19.0)	1.27 (0.91-1.77)		618 (62.1)	1.15 (0.77-1.72)	

Adjusted household income model									
Highest	83 (16.2)	1.00	n.s.	349 (35.6)	1.00	0.05	117 (15.7)	1.00	0.17
2nd highest	145 (28.3)	0.70 (0.39-1.23)		269 (27.4)	1.12 (0.81-1.50)		160 (21.5)	0.85 (0.52-1.40)	
2nd lowest	149 (29.1)	1.18 (0.67-2.06)		177 (18.0)	1.26 (0.87-1.83)		234 (31.4)	0.96 (0.60-1.54)	
Lowest	135 (26.4)	0.83 (0.48-1.46)		187 (19.0)	1.34 (0.96-1.86)		235 (31.5)	1.20 (0.77-1.88)	

* : all ORs were adjusted by age, gender, marital status, and area.

† : p value for trend test

## DISCUSSION

The primary objective of this research was to examine the existence of social gradients in health among the general population in Japan. The results of our study indicated a gradient association between self-rated physical and mental health with levels of educational attainment. The respondents with lower levels of educational attainment were more likely to rate their physical and mental health as poor than those with higher educational attainment.

U or J-shaped associations were found to exist between adjusted household income and self-rated physical and mental health for all respondents (Table 2), between adjusted household income and mental health for males (Table 2), and between adjusted household income and self-rated physical health for female workers (Table 3). A recent study in Japan reported that leisure-time physical activity, a health-related habit, was less among both higher-class and lower-class occupations, indicating a reversed U-shaped association between occupational class and health-related behaviors in Japan.^35^ A greater commitment to work and longer overtime hours among those who earned high incomes and were employed in high-class occupations, such as managers, may cause poor physical and mental health status in Japan.

Overall, our results suggested that educational attainment was a better predictor of self-rated health than adjusted household income. Shibuya et al^26^ showed that adjusted household income was a strong predictor of self-rated health, controlling regional income inequalities in Japan. One of the possible reasons for the inconsistency between our results and those of Shibuya et al might be differences in the study population. They used random samples of the general population throughout Japan, while we used random samples from selected areas, which did not include urban cities.

Our secondary objective in conducting this research was to examine gender differences related to social inequalities in self-rated health. We identified significant social inequalities in self-rated physical and mental health only among female respondents. Our results were somewhat inconsistent with previously conducted studies. Martikainen et al^20^ showed the association between self-rated health and employment grade among 40- to 60-year-old Japanese male employees in selected cities, but they indicated small or inconsistent social inequalities in self-rated health among Japanese female employees. One possible reason for this inconsistency with our results could be the difference in indicators of social class. They used employment grade as a social class indicator. As they noted, their indicator could not be a relevant measure of female social class, and therefore they suggested that a household-based measure could be a better indicator for females. On the other hand, it is possible that their indicator is a better measure for males than educational attainment and household income, especially for middle-aged males. Unfortunately, we did not use employment or occupational classification as an indicator of social class in the present study. A further study should directly test this hypothesis using multiple measures of social class, education, income, and occupation.

A previously conducted study by Nishi et al^21^ showed an inverse association of self-rated health with employment grade in both male and female Japanese civil servants aged 35 years or older in a city. They also found an inverse association of self-rated health with education, but only in male employees. This discrepancy with our results could be due to differences in the study populations. Their study population comprised civil servants in an urban city, while ours consisted of the general population from selected areas, excluding urban cities. In addition, because their study subjects were all civil servants, the range of social classes that they captured could have been narrower than that of our study population.

The results of the stratified analysis by gender and employment situation indicated that female workers have significant social inequalities in self-rated physical and mental health. We also saw some associations between educational attainment level and self-rated physical and mental health among housewives. However, housewives had no associations between adjusted household income level and self-rated mental health. The effect of multiple social roles on self-rated mental health could be one explanation for the greater social inequalities in self-rated mental health among female workers than among housewives. According to national polls conducted in 2000, 84.9% of two-income couples reported that the wife was responsible for daily household affairs such as cleaning, washing, and cooking.^36^ Multiple social roles are thought to affect an individual’s health in two ways: role overload and role enhancement.^37^ From the former perspective, female multiple-role experiences are likely to lead to role overload and conflict, which result in poor physical and mental health. From the latter perspective, female multiple-role experiences could benefit and enhance physical and mental health. Previous research suggests that the health enhancing effect of multiple-role experiences among females is less clear or even absent for lower social classes, while it is more prominent among higher social classes.^38^ The functioning of multiple social roles, therefore, could be different depending on social class, and household financial conditions could be an important factor in determining the function of multiple social roles. Further longitudinal studies will be required to examine the mechanism of inequalities in health among Japanese females.

We identified a significant association of educational attainment with self-rated physical health and a gradient association of educational attainment with self-rated mental health among respondents 20 to 40 years of age. On the other hand, no associations were found between adjusted household income and self-rated health. Educational attainment, therefore, could be a better social class indicator than household income for younger generations. Among middle-aged respondents, there was a gap found with respect to physical and mental health by educational attainment level, although the associations were not clearly linear. Adjusted household income had a significant association with self-rated physical health, and it seemed to have an inverse association with mental health. One reason that household income seemed to be a more significant predictor of self-rated health than educational attainment in this group was, as previous studies have indicated,^20^^,^^21^ that employment grade may be an important indicator in a middle-aged population and that household income may link to employment grade more closely than educational attainment. Among those 61 years old and older, adjusted household income showed a significant association with self-rated physical health. Those in the lowest income group were at significantly higher risk of having poor self-rated physical health, compared with those in the highest household income group. The other associations between social class and self-rated heath seemed to be either U- or J-shaped, although they were not significant.

Our data suggested that social inequalities exist across all age groups. However, the appropriate indicators for social class may be different depending on the age group; educational attainment may be a better social class indicator for a younger generation, while adjusted household income may be a better indicator for older generations. The different results among age groups suggest differences in the mechanisms of social inequalities in self-rated health, that is, how social class influences self-rated health across age groups. For example, among young respondents, differences in their future prospects as a result of their educational attainment levels could affect self ratings for physical and mental health, while among older respondents material conditions based on their financial situation could be the most important factor in influencing self rated health.

There are several limitations in this study. First, the results may be limited by the use of a biased sample. We used random samples from only selected sites, mainly from western Japan, and did not include a metropolitan city. Second, the relatively low response rate (58.5%) may also limit the interpretation of our results. Subjects who did not complete the survey were likely to be younger than our study sample and likely to be men, which may lead to a potential bias due to non-response. Third, our study was also limited by weak measurement of social class. Assessment of social class is quite complex.^9^^,^^39^ Although we used two social class indicators, imprecise measurements of social class could have distorted the association between social class and self-rated health. Additional measures of social class (such as occupation and wealth) would have increased the reliability of our findings. Fourth, we used an interview survey to measure self-rated health, which may lead to somewhat different results from those obtained by studies using self-administrated questionnaires. Finally, although we showed differences in self-rated health status across social class, we need to be cautious about inferring a causal association between social class and health status from our cross-sectional study. The mechanism of reverse causation could be possible, especially for adjusted household income and health.

In summary, the results of this study imply discrepancies in self-rated physical and mental health along lines of social class in Japan. Japanese females disproportionately experienced social inequalities in self-rated health. In addition, although we identified social inequalities in self-rated health across all age groups, differences in the respective mechanisms of social inequalities in self-rated health were suggested.
